# Non-idempotent Intersection Types in Logical Form

**DOI:** 10.1007/978-3-030-45231-5_11

**Published:** 2020-04-17

**Authors:** Thomas Ehrhard

**Affiliations:** 8 ENS/CNRS, Université Paris-Saclay, Cachan, France; 9 University of Duisburg-Essen, Duisburg, Germany; Université de Paris, IRIF, CNRS, 75013 Paris, France

**Keywords:** Lambda Calculus, Denotational Semantics, Intersection Types, Linear Logic

## Abstract

Intersection types are an essential tool in the analysis of operational and denotational properties of lambda-terms and functional programs. Among them, non-idempotent intersection types provide precise quantitative information about the evaluation of terms and programs. However, unlike simple or second-order types, intersection types cannot be considered as a logical system because the application rule (or the intersection rule, depending on the presentation of the system) involves a condition stipulating that the proofs of premises must have the same structure. Using earlier work introducing an indexed version of Linear Logic, we show that non-idempotent typing can be given a logical form in a system where formulas represent hereditarily indexed families of intersection types.

## Introduction

Intersection types, introduced in the work of Coppo and Dezani [4,5] and developed since then by many authors, are still a very active research topic. As quite clearly explained in [13], the Coppo and Dezani intersection type system $D\Omega$ can be understood as a syntactic presentation of the denotational interpretation of $\lambda$-terms in the Engeler's model, which is a model of the pure $\lambda$-calculus in the cartesian closed category of prime-algebraic complete lattices and Scott continuous functions.

Intersection types can be considered as formulas of the propositional calculus with implication $\Rightarrow$ and conjunction $\wedge$ as connectives. However, as pointed out by Hindley [12], intersection types deduction rules depart drastically from the standard logical rules of intuitionistic logic (and of any standard logical system) by the fact that, in the $\wedge$-introduction rule, it is assumed that the proofs of the two premises are typings of the *same* $\lambda$-term, which means that, in some sense made precise by the typing system itself, they have the same structure. Such requirements on *proofs* premises, and not only on formulas proven in premises,

* Partially supported by the project ANR-19-CE48-0014 PPS.

are absent from standard (intuitionistic or classical) logical systems where the proofs of premises are completely independent from each other. Many authors have addressed this issue, we refer to [14] for a discussion on several solutions which mainly focus on the design of *à la Church* presentations of intersection typing systems, thus enriching $\lambda$-terms with additional structures. Among the most recent and convincing contributions to this line of research we should certainly mention [15].

In our "new" approach to this problem — not so new actually since it dates back to [3] —, we change formulas instead of changing terms. It is based on a specific model of Linear Logic (and thus of the $\lambda$-calculus): the *relational model*. It is fair to credit Girard for the introduction of this model since it appears at least implicitly in [11]. It was probably known by many people in the Linear Logic community as a piece of folklore since the early 1990's and is presented formally in [3]. In this quite simple and canonical denotational model, types are interpreted as sets (without any additional structure) and a closed term of type $\sigma$ is interpreted as a subset of the interpretation of $\sigma$. It is quite easy to define, in this semantic framework, analogues of the usual models of the pure $\lambda$-calculus such as Scott's $D_\infty$ or Engeler's model, which in some sense are simpler than the original ones since the sets interpreting types need not to be pre-ordered. As explained in the work of De Carvalho [6,7], the intersection type counterpart of this semantics is a typing system where "intersection" is non-idempotent (in sharp contrast with the original systems introduced by Coppo and Dezani), sometimes called *system R*. Notice that the precise connection between the idempotent and non-idempotent approaches is analyzed in [8], in a quite general Linear Logic setting by means of an extensional collapse.

In order to explain our approach, we restrict first to simple types, interpreted as follows in the relational model: a basic type $\alpha$ is interpreted as a given set $[\![\alpha]\!]$ and the type $\sigma \Rightarrow \tau$ is interpreted as the set $\mathcal{M}_{\mathrm{fin}}([\![\sigma]\!]) \times [\![\tau]\!]$ (where $\mathcal{M}_{\mathrm{fin}}(E)$ is the set of finite multisets of elements of $E$). Remember indeed that intersection types can be considered as a syntactic presentation of denotational semantics, so it makes sense to define intersection types relative to simple types (in the spirit of [10]) as we do in Section 3: an intersection type relative to the base type $\alpha$ is an element of $[\![\alpha]\!]$ and an intersection type relative to $\sigma \Rightarrow \tau$ is a pair $([\,a_1, \dots, a_n\,], b)$ where the $a_i$s are intersection types relative to $\sigma$ and $b$ is an intersection type relative to $\tau$; with more usual notations[1] $([\,a_1, \dots, a_n\,], b)$ would be written $(a_1 \wedge \cdots \wedge a_n) \rightarrow b$. Then, given a type $\sigma$, the main idea consists in representing an indexed family of elements of $[\![\sigma]\!]$ as a formula of a new logical system. If $\sigma = (\varphi \Rightarrow \psi)$ then the family can be written[2] $([\,a_k \mid k \in K \text{ and } u(k) = j\,], b_j)_{j \in J}$ where $J$ and $K$ are indexing sets, $u : K \to J$ is a function such that $f^{-1}(\{j\})$ is finite for all $j \in J$, $(b_j)_{j \in J}$ is a family of elements of $[\![\psi]\!]$ (represented by a formula $B$) and $(a_k)_{k \in K}$ is a family of elements of $[\![\varphi]\!]$ (represented by a formula $A$): in that case we introduce the implicative formula $(A \Rightarrow_u B)$ to represent the family

[1] That we prefer not to use for avoiding confusions between these two levels of typing.
[2] We use $[\cdots]$ for denoting multisets much as one uses $\{\cdots\}$ for denoting sets, the only difference is that multiplicities are taken into account.

$([\, a_k \mid k \in K \text{ and } u(k) = j\,], b_j)_{j\in J}$. It is clear that a family of simple types has generally infinitely many representations as such formulas; this huge redundancy makes it possible to establish a tight link between inhabitation of intersection types with provability of formulas representing them (in an indexed version $\mathsf{LJ}(I)$ of intuitionistic logic). Such a correspondence is exhibited in Section 3 in the simply typed setting and the idea is quite simple:

> given a type $\sigma$, a family $(a_j)_{j\in J}$ of elements of $[\![\sigma]\!]$, and a closed $\lambda$-term of type $\sigma$, it is equivalent to say that $\vdash M : a_j$ holds for all $j$ and to say that some (and actually any) formula $A$ representing $(a_j)_{j\in J}$ has an $\mathsf{LJ}(I)$ proof[3] whose underlying $\lambda$-term is $M$.

In Section 4 we extend this approach to the untyped $\lambda$-calculus taking as underlying model of the pure $\lambda$-calculus our relational version $\mathsf{R}_\infty$ of Scott's $D_\infty$. We define an adapted version of $\mathsf{LJ}(I)$ and establish a similar correspondence, with some slight modifications due to the specificities of $\mathsf{R}_\infty$.

## 1 Notations and preliminary definitions

If $E$ is a set, a *finite multiset of elements of* $E$ is a function $m : E \to \mathbb{N}$ such that the set $\{a \in E \mid m(a) \neq 0\}$ (called the *domain* of $m$) is finite. The cardinal of such a multiset $m$ is $\#m = \sum_{a\in E} m(a)$. We use $+$ for the obvious addition operation on multisets, and if $a_1, \dots, a_n$ are elements of $E$, we use $[\, a_1, \dots, a_n\,]$ for the corresponding multiset (taking multiplicities into account); for instance $[\,0, 1, 0, 2, 1\,]$ is the multiset $m$ of elements of $\mathbb{N}$ such that $m(0) = 2$, $m(1) = 2$, $m(2) = 1$ and $m(i) = 0$ for $i > 2$. If $(a_i)_{i\in I}$ is a family of elements of $E$ and if $J$ is a finite subset of $I$, we use $[\, a_i \mid i \in J\,]$ for the multiset of elements of $E$ which maps $a \in E$ to the number of elements $i \in J$ such that $a_i = a$ (which is finite since $J$ is). We use $\mathcal{M}_{\mathrm{fin}}(E)$ for the set of finite multisets of elements of $E$.

We use $+$ to denote set union when we we want to stress the fact that the involved sets are disjoint. A function $u : J \to K$ is *almost injective* if $\#u^{-1}\{k\}$ is finite for each $k \in K$ (equivalently, the inverse image of any finite subset of $K$ under $u$ is finite). If $s = (a_1, \dots, a_n)$ is a sequence of elements of $E$ and $i \in \{1, \dots, n\}$, we use $(s) \setminus i$ for the sequence $(a_1, \dots, a_{i-1}, a_{i+1}, \dots, a_n)$. Given sets $E$ and $F$, we use $F^E$ for the set of function from $E$ to $F$. The elements of $F^E$ are sometimes considered as functions $u$ (with a functional notation $u(e)$ for application) and sometimes as indexed families $a$ (with index notations $a_e$ for application) especially when $E$ is countable.

If $i \in \{1, \dots, n\}$ and $j \in \{1, \dots, n-1\}$, we define $\mathsf{s}(j, i) \in \{1, \dots, n\}$ as follows: $\mathsf{s}(j, i) = j$ if $j < i$ and $\mathsf{s}(j, i) = j + 1$ if $j \geq i$.

[3] Any such proof can be stripped from its indexing data giving rise to a proof of $\sigma$ in intuitionistic logic.

## 2 The relational model of the $\lambda$-calculus

Let $\mathbf{Rel}_!$ the category whose objects are sets[4] and $\mathbf{Rel}_!(X,Y) = \mathcal{P}(\mathcal{M}_{\mathrm{fin}}(X) \times Y)$ with $\mathsf{Id}_X = \{([a],a) \mid a \in X\}$ and composition of $s \in \mathbf{Rel}_!(X,Y)$ and $t \in \mathbf{Rel}_!(Y,Z)$ given by

$$t \circ s = \{(m_1 + \cdots + m_k, c) \mid \\ \exists b_1,\dots,b_k \in Y\ ([b_1,\dots,b_k],c) \in t \text{ and } \forall j\ (m_j,b_j) \in s\}\,.$$

It is easily checked that this composition law is associative and that $\mathsf{Id}$ is neutral for composition[5]. This category has all countable products: let $(X_j)_{j\in J}$ be a countable family of sets, their product is $X = \&_{j\in J} X_j = \bigcup_{j\in J}\{j\} \times X_j$ and projections $(\mathsf{pr}_j)_{j\in J}$ given by $\mathsf{pr}_j = \{([(j,a)],a) \mid a \in X_j\} \in \mathbf{Rel}_!(X,X_j)$ and if $(s_j)_{j\in J}$ is a family of morphisms $s_j \in \mathbf{Rel}_!(Y,X_j)$ then their tupling is $\langle s_j\rangle_{j\in J} = \{([a],(j,b))) \mid j \in J \text{ and } ([a],b) \in s_j\} \in \mathbf{Rel}_!(Y,X)$.

The category $\mathbf{Rel}_!$ is cartesian closed with object of morphisms from $X$ to $Y$ the set $(X \Rightarrow Y) = \mathcal{M}_{\mathrm{fin}}(X) \times Y$ and evaluation morphism $\mathsf{Ev} \in \mathbf{Rel}_!((X \Rightarrow Y) \,\&\, X, Y)$ is given by $\mathsf{Ev} = \{([(1,[a_1,\dots,a_k],b),(2,a_1),\dots,(2,a_k)],b) \mid a_1,\dots,a_k \in X \text{ and } b \in Y\}$. The transpose (or curryfication) of $s \in \mathbf{Rel}_!(Z \,\&\, X, Y)$ is $\mathsf{Cur}(s) \in \mathbf{Rel}_!(Z, X \Rightarrow Y)$ given by $\mathsf{Cur}(s) = \{([c_1,\dots,c_n],([a_1,\dots,a_k],b)) \mid ([(1,c_1),\dots,(1,c_n),(2,a_1),\dots,(2,a_k)],c) \in s\}$.

**Relational $D_\infty$.** Let $\mathsf{R}_\infty$ be the least set such that $(m_0,m_1,\dots) \in \mathsf{R}_\infty$ as soon as $m_0,m_1\dots$ are finite multisets of elements of $\mathsf{R}_\infty$ which are almost all equal to $[\,]$. Notice in particular that $\mathsf{e} = ([\,],[\,],\dots) \in \mathsf{R}_\infty$ and satisfies $\mathsf{e} = ([\,],\mathsf{e})$. By construction we have $\mathsf{R}_\infty = \mathcal{M}_{\mathrm{fin}}(\mathsf{R}_\infty) \times \mathsf{R}_\infty$, that is $\mathsf{R}_\infty = (\mathsf{R}_\infty \Rightarrow \mathsf{R}_\infty)$ and hence $\mathsf{R}_\infty$ is a model of the pure $\lambda$-calculus in $\mathbf{Rel}_!$ which also satisfies the $\eta$-rule. See [1] for general facts on this kind of model.

## 3 The simply typed case

We assume to be given a set of type atoms $\alpha,\beta,\dots$ and of variables $x,y,\dots$; types and terms are given as usual by $\sigma,\tau,\dots := \alpha \mid \sigma \Rightarrow \tau$ and $M,N,\dots := x \mid (M)\,N \mid \lambda x^\sigma\, N$.

With any type atom we associate a set $[\![\alpha]\!]$. This interpretation is extended to all types by $[\![\sigma \Rightarrow \tau]\!] = [\![\sigma]\!] \Rightarrow [\![\tau]\!] = \mathcal{M}_{\mathrm{fin}}([\![\sigma]\!]) \times [\![\tau]\!]$. The relational semantics of this $\lambda$-calculus can be described as a non-idempotent intersection type system, with judgments of shape $x_1 : m_1 : \sigma_1,\dots,x_n : m_n : \sigma_n \vdash M : a : \sigma$ where the $x_i$'s are pairwise distinct variables, $M$ is a term, $a \in [\![\sigma]\!]$ and $m_i \in \mathcal{M}_{\mathrm{fin}}([\![\sigma_i]\!])$ for each $i$. Here are the typing rules:

$$\frac{j \neq i \Rightarrow m_j = [\,] \text{ and } m_i = [a]}{(x_i : m_i : \sigma_i)_{i=1}^n \vdash x_i : a : \sigma} \qquad \frac{\Phi, x : m : \sigma \vdash M : b : \tau}{\Phi \vdash \lambda x^\sigma\, M : (m,b) : \sigma \Rightarrow \tau}$$

[4] We can restrict to countable sets.

[5] This results from the fact that $\mathbf{Rel}_!$ arises as the Kleisli category of the $\mathsf{LL}$ model of sets and relations, see [3] for instance.

$$\frac{\Phi \vdash M : ([a_1,\dots,a_k],b) : \sigma \Rightarrow \tau \qquad (\Phi_l \vdash N : a_l : \sigma)_{l=1}^{k}}{\Psi \vdash (M)\,N : b : \tau}$$

where $\Phi = (x_i : m_i : \sigma_i)_{i=1}^n$, $\Phi_l = (x_i : m_i^l : \sigma_i)_{i=1}^n$ for $l = 1,\dots,k$ and $\Psi = (x_i : m_i + \sum_{l=1}^k m_i^l : \sigma_i)_{i=1}^n$.

### 3.1 Why do we need another system?

The trouble with this deduction system is that it cannot be considered as the term decorated version of an underlying "logical system for intersection types" allowing to prove sequents of shape $m_1 : \sigma_1,\dots,m_n : \sigma_n \vdash a : \sigma$ (where non-idempotent intersection types $m_i$ and $a$ are considered as logical formulas, the ordinary types $\sigma_i$ playing the role of "kinds") because, in the application rule above, it is required that all the proofs of the $k$ right hand side premises have the same shape given by the $\lambda$-term $N$. We propose now a "logical system" derived from [3] which, in some sense, solves this issue. The main idea is quite simple and relies on three principles: (1) replace *hereditarily* multisets with indexed families in intersection types, (2) instead of proving single types, prove indexed families of hereditarily indexed types and (3) represent syntactically such families (of hereditarily indexed types) as formulas of a new system of *indexed logic*.

### 3.2 Minimal $\mathsf{LJ}(I)$

We define now the syntax of indexed formulas. Assume to be given an infinite countable set $I$ of indices. Then we define indexed types $A$; with each such type we associate an underlying type $\underline{A}$, a set $\mathsf{d}(A)$ and a family $\langle A\rangle \in [\![\underline{A}]\!]^{\mathsf{d}(A)}$. These formulas are given by the following inductive definition:

- if $J \subseteq I$ and $f : J \to [\![\alpha]\!]$ is a function then $\alpha[f]$ is a formula with $\underline{\alpha[f]} = \alpha$, $\mathsf{d}(\alpha[f]) = J$ and $\langle \alpha[f]\rangle = f$
- and if $A$ and $B$ are formulas and $u : \mathsf{d}(A) \to \mathsf{d}(B)$ is almost injective then $A \Rightarrow_u B$ is a formula with $\underline{A \Rightarrow_u B} = \underline{A} \Rightarrow \underline{B}$, $\mathsf{d}(A \Rightarrow_u B) = \mathsf{d}(B)$ and, for $k \in \mathsf{d}(B)$, $\langle A \Rightarrow_u B\rangle_k = ([\,\langle A\rangle_j \mid j \in \mathsf{d}(A) \text{ and } u(j) = k\,], \langle B\rangle_k)$.

**Proposition 1.** *Let $\sigma$ be a type, $J$ be a subset of $I$ and $f \in [\![\sigma]\!]^J$. There is a formula $A$ such that $\underline{A} = \sigma$, $\mathsf{d}(A) = J$ and $\langle A\rangle = f$ (actually, there are infinitely many such $A$'s as soon as $\sigma$ is not an atom and $J \neq \emptyset$).*

*Proof.* The proof is by induction on $\sigma$. If $\sigma$ is an atom $\alpha$ then we take $A = \alpha[f]$. Assume that $\sigma = (\rho \Rightarrow \tau)$ so that $f(j) = (m_j, b_j)$ with $m_j \in \mathcal{M}_{\mathrm{fin}}([\![\rho]\!])$ and $b_j \in [\![\tau]\!]$. Since each $m_j$ is finite and $I$ is infinite, we can find a family $(K_j)_{j\in J}$ of pairwise disjoint finite subsets of $I$ such that $\#K_j = \#m_j$. Let $K = \bigcup_{j\in J} K_j$, there is a function $g : K \to [\![\rho]\!]$ such that $m_j = [\,g(k) \mid k \in K_j\,]$ for each $j \in J$ (choose first an enumeration $g_j : K_j \to [\![\rho]\!]$ of $m_j$ for each $j$ and then define $g(k) = g_j(k)$ where $j$ is the unique element of $J$ such that $k \in K_j$). Let $u : K \to J$ be the unique function such that $k \in K_{u(k)}$ for all $k \in K$; since each $K_j$ is finite, this function $u$ is almost injective. By inductive hypothesis there is a formula $A$ such that $\underline{A} = \rho$, $\mathsf{d}(A) = K$ and $\langle A \rangle = g$, and there is a formula $B$ such that $\underline{B} = \tau$, $\mathsf{d}(B) = J$ and $\langle B \rangle = (b_j)_{j \in J}$. Then the formula $A \Rightarrow_u B$ is well formed (since $u$ is an almost injective function $\mathsf{d}(A) = K \to \mathsf{d}(B) = J$) and satisfies $\underline{A \Rightarrow_u B} = \sigma$, $\mathsf{d}(A \Rightarrow_u B) = J$ and $\langle A \Rightarrow_u B \rangle = f$ as contended. □

As a consequence, for any type $\sigma$ and any element $a$ of $[\![\sigma]\!]$ (so $a$ is a non-idempotent intersection type of kind $\sigma$), one can find a formula $A$ such that $\underline{A} = \sigma$, $\mathsf{d}(A) = \{j\}$ (where $j$ is an arbitrary element of $I$) and $\langle A \rangle_j = a$. In other word, any intersection type can be represented as a formula (in infinitely many different ways in general of course, but up to renaming of indices, that is, up to "hereditary $\alpha$-equivalence", this representation is unique).

For any formula $A$ and $J \subseteq I$, we define a formula $A\!\upharpoonright_J$ such that $\underline{A\!\upharpoonright_J} = \underline{A}$, $\mathsf{d}(A\!\upharpoonright_J) = \mathsf{d}(A) \cap J$ and $\langle A\!\upharpoonright_J \rangle = \langle A \rangle \upharpoonright_J$. The definition is by induction on $A$.

- $\alpha[f]\!\upharpoonright_J = \alpha[f \upharpoonright_J]$
- $(A \Rightarrow_u B)\!\upharpoonright_J = (A\!\upharpoonright_K \Rightarrow_v B\!\upharpoonright_J)$ where $K = u^{-1}(\mathsf{d}(B) \cap J)$ and $v = u \upharpoonright_K$.

Let $u : \mathsf{d}(A) \to J$ be a *bijection* (so that $u(\mathsf{d}(A)) = J$), we define a formula $u_*(A)$ such that $\underline{u_*(A)} = \underline{A}$, $\mathsf{d}(u_*(A)) = u(\mathsf{d}(A))$ and $\langle u_*(A) \rangle_j = \langle A \rangle_{u^{-1}(j)}$. The definition is by induction on $A$:

- $u_*(\alpha[f]) = \alpha[f \circ u^{-1}]$
- $u_*(A \Rightarrow_v B) = (A \Rightarrow_{u \circ v} u_*(B))$.

Using these two auxiliary notions, we can give a set of three deduction rules for a minimal natural deduction allowing to prove formulas in this indexed intuitionistic logic. This logical system allows to derive sequents which are of shape

$$A_1^{u_1}, \ldots, A_n^{u_n} \vdash B \tag{1}$$

where for each $i = 1, \ldots, n$, the function $u_i : \mathsf{d}(A_i) \to \mathsf{d}(B)$ is almost injective (it is not required that $\mathsf{d}(B) = \bigcup_{i=1}^n u_i(\mathsf{d}(A_i))$). Notice that the expressions $A_i^{u_i}$ are not formulas; this construction $A^u$ is part of the syntax of sequents, just as the "," separating these pseudo-formulas. Given a formula $A$ and $u : \mathsf{d}(A) \to J$ almost injective, it is nevertheless convenient to define $\langle A^u \rangle \in \mathcal{M}_{\mathrm{fin}}([\![\underline{A}]\!])^J$ by $\langle A^u \rangle_j = [\, \langle A \rangle_k \mid u(k) = j \,]$. In particular, when $u$ is a bijection, $\langle A^u \rangle_j = [\, \langle A \rangle_{u^{-1}(j)} \,]$.

The crucial point here is that such a sequent (1) involves no $\lambda$-term.

The main difference between the original system $\mathsf{LL}(I)$ of [3] and the present system is the way axioms are dealt with. In $\mathsf{LL}(I)$ there is no explicit identity axiom and only "atomic axioms" restricted to the basic constants of $\mathsf{LL}$; indeed it is well-known that in $\mathsf{LL}$ all identity axioms can be $\eta$-expanded, leading to proofs using only such atomic axioms. In the $\lambda$-calculus, and especially in the untyped $\lambda$-calculus we want to deal with in next sections, such $\eta$-expansions are hard to handle so we prefer to use explicit identity axioms.

The axiom is

$$\frac{j \neq i \Rightarrow \mathsf{d}(A_j) = \emptyset \text{ and } u_i \text{ is a bijection}}{A_1^{u_1}, \ldots, A_n^{u_n} \vdash u_{i*}(A_i)}$$

so that for $j \neq i$, the function $u_j$ is empty. A special case is

$$\frac{j \neq i \Rightarrow \mathsf{d}(A_j) = \emptyset \text{ and } u_i \text{ is the identity function}}{A_1^{u_1}, \dots, A_n^{u_n} \vdash A_i}$$

which may look more familiar, but the general axiom rule, allowing to "delocalize" the proven formula $A_i$ by an arbitrary bijection $u_i$, is required as we shall see. The $\Rightarrow$ introduction rule is quite simple

$$\frac{A_1^{u_1}, \dots, A_n^{u_n}, A^u \vdash B}{A_1^{u_1}, \dots, A_n^{u_n} \vdash A \Rightarrow_u B}$$

Last the $\Rightarrow$ elimination rule is more complicated (from a Linear Logic point of view, this is due to the fact that it combines 3 $\mathsf{LL}$ logical rules: $\multimap$ elimination, contraction and promotion). We have the deduction

$$\frac{C_1^{u_1}, \dots, C_n^{u_n} \vdash A \Rightarrow_u B \qquad D_1^{v_1}, \dots, D_n^{v_n} \vdash A}{E_1^{w_1}, \dots, E_n^{w_n} \vdash B}$$

under the following conditions, to be satisfied by the involved formulas and functions: for each $i = 1, \dots, n$ one has $\mathsf{d}(C_i) \cap \mathsf{d}(D_i) = \emptyset$, $\mathsf{d}(E_i) = \mathsf{d}(C_i) + \mathsf{d}(D_i)$, $C_i = E_i \restriction_{\mathsf{d}(C_i)}$, $D_i = E_i \restriction_{\mathsf{d}(D_i)}$, $w_i \restriction_{\mathsf{d}(C_i)} = u_i$, and $w_i \restriction_{\mathsf{d}(D_i)} = u \circ v_i$.

Let $\pi$ be a deduction tree of the sequent $A_1^{u_1}, \dots, A_n^{u_n} \vdash B$ in this system. By dropping all index information we obtain a derivation tree $\underline{\pi}$ of $\underline{A_1}, \dots, \underline{A_n} \vdash \underline{B}$, and, upon choosing a sequence $\overrightarrow{x}$ of $n$ pairwise distinct variables, we can associate with this derivation tree a simply typed $\lambda$-term $\underline{\pi}_{\overrightarrow{x}}$ which satisfies $x_1 : \underline{A_1}, \dots, x_n : \underline{A_n} \vdash \underline{\pi}_{\overrightarrow{x}} : \underline{B}$.

### 3.3 Basic properties of $\mathsf{LJ}(I)$

We prove some basic properties of this logical system. This is also the opportunity to get some acquaintance with it. Notice that in many places we drop the type annotations of variables in $\lambda$-terms, first because they are easy to recover, and second because the very same results and proofs are also valid in the untyped setting of Section 4.

**Lemma 1 (Weakening).** *Assume that $\Phi \vdash A$ is provable by a proof $\pi$ and let $B$ be a formula such that $\mathsf{d}(B) = \emptyset$. Then $\Phi' \vdash A$ is provable by a proof $\pi'$, where $\Phi'$ is obtained by inserting $B^{0_{\mathsf{d}(A)}}$ at any place in $\Phi$. Moreover $\underline{\pi}_{\overrightarrow{x}} = \underline{\pi'}_{\overrightarrow{x'}}$ (where $\overrightarrow{x'}$ is obtained from $\overrightarrow{x}$ by inserting a dummy variable at the same place).*

The proof is an easy induction on the proof of $\Phi \vdash A$.

**Lemma 2 (Relocation).** *Let $\pi$ be a proof of $(A_i^{u_i})_{i=1}^n \vdash A$ let $u : \mathsf{d}(A) \to J$ be a bijection, there is a proof $\pi'$ of $(A_i^{u \circ u_i})_{i=1}^n \vdash u_*(A)$ such that $\underline{\pi'}_{\overrightarrow{x}} = \underline{\pi}_{\overrightarrow{x}}$.*

The proof is a straightforward induction on $\pi$.

**Lemma 3 (Restriction).** *Let $\pi$ be a proof of $(A_i^{u_i})_{i=1}^n \vdash A$ and let $J \subseteq \mathsf{d}(A)$. For $i = 1, \dots, n$, let $K_i = u_i{}^{-1}(J) \subseteq \mathsf{d}(A_i)$ and $u_i' = u_i \restriction_{K_i} : K_i \to J$. Then the sequent $((A_i \restriction_{K_i})^{u_i'})_{i=1}^n \vdash A \restriction_J$ has a proof $\pi'$ such that $\underline{\pi'}_{\overrightarrow{x}} = \underline{\pi}_{\overrightarrow{x}}$.*

*Proof.* By induction on $\pi$. Assume that $\pi$ consists of an axiom $(A_j^{u_j})_{j=1}^n \vdash u_{i*}(A_i)$ with $\mathsf{d}(A_j) = \emptyset$ if $j \neq i$, and $u_i$ a bijection. With the notations of the lemma, $K_j = \emptyset$ for $j \neq i$ and $u'_i$ is a bijection $K_i \to J$. Moreover $u'_{i*}(A_i\upharpoonright_{K_i}) = u_{i*}(A_i)\upharpoonright_J$ so that $((A_i\upharpoonright_{K_i})^{u'_i})_{i=1}^n \vdash A\upharpoonright_J$ is obtained by an axiom $\pi'$ with $\underline{\pi'}_{\vec{x}} = x_i = \underline{\pi}_{\vec{x}}$.

Assume that $\pi$ ends with a $\Rightarrow$-introduction rule:

$$\dfrac{\begin{array}{c}\rho \\ (A_i^{u_i})_{i=1}^{n+1} \vdash B\end{array}}{(A_i^{u_i})_{i=1}^{n} \vdash A_{n+1} \Rightarrow_{u_{n+1}} B}$$

with $A = (A_{n+1} \Rightarrow_{u_{n+1}} B)$, and we have $\underline{\pi}_{\vec{x}} = \lambda x_{n+1}\, \underline{\rho}_{\vec{x},x_{n+1}}$. With the notations of the lemma we have $A\upharpoonright_J = (A_{n+1}\upharpoonright_{K_{n+1}} \Rightarrow_{u'_{n+1}} B\upharpoonright_J)$. By inductive hypothesis there is a proof $\rho'$ of $(A_i\upharpoonright_{K_i}^{u'_i})_{i=1}^{n+1} \vdash B\upharpoonright_J$ such that $\underline{\rho'}_{\vec{x},x_{n+1}} = \underline{\rho}_{\vec{x},x_{n+1}}$ and hence we have a proof $\pi'$ of $(A_i\upharpoonright_{K_i}^{u'_i})_{i=1}^{n} \vdash A\upharpoonright_J$ with $\underline{\pi'}_{\vec{x}} = \lambda x_{n+1}\, \underline{\rho'}_{\vec{x},x_{n+1}} = \underline{\pi}_{\vec{x}}$ as contended.

Assume last that $\pi$ ends with a $\Rightarrow$-elimination rule:

$$\dfrac{\begin{array}{c}\mu \\ (B_i^{v_i})_{i=1}^{n} \vdash B \Rightarrow_v A\end{array} \qquad \begin{array}{c}\rho \\ (C_i^{w_i})_{i=1}^{n} \vdash B\end{array}}{(A_i^{u_i})_{i=1}^{n} \vdash A}$$

with $\mathsf{d}(A_i) = \mathsf{d}(B_i) + \mathsf{d}(C_i)$, $B_i = A_i\upharpoonright_{\mathsf{d}(B_i)}$ and $C_i = A_i\upharpoonright_{\mathsf{d}(C_i)}$, $u_i\upharpoonright_{\mathsf{d}(B_i)} = v_i$ and $u_i\upharpoonright_{\mathsf{d}(C_i)} = v \circ w_i$ for $i = 1, \dots, n$, and of course $\underline{\pi}_{\vec{x}} = \left(\underline{\mu}_{\vec{x}}\right) \underline{\rho}_{\vec{x}}$. Let $L = v^{-1}(J) \subseteq \mathsf{d}(B)$. Let $L_i = {v_i}^{-1}(J)$ and $R_i = {w_i}^{-1}(L)$ for $i = 1, \dots, n$ (we also set $v'_i = v_i\upharpoonright_{L_i}$, $w'_i = w_i\upharpoonright_{R_i}$ and $v' = v\upharpoonright_L$). By inductive hypothesis, we have a proof $\mu'$ of $(B_i\upharpoonright_{L_i}^{v'_i})_{i=1}^n \vdash B\upharpoonright_L \Rightarrow_{v'} A\upharpoonright_J$ such that $\underline{\mu'}_{\vec{x}} = \underline{\mu}_{\vec{x}}$ and a proof $\rho'$ of $(C_i\upharpoonright_{R_i}^{w'_i})_{i=1}^n \vdash B\upharpoonright_L$ such that $\underline{\rho'}_{\vec{x}} = \underline{\rho}_{\vec{x}}$. Now, setting $K_i = {u_i}^{-1}(K)$, observe that

- $\mathsf{d}(B_i) \cap K_i = L_i = \mathsf{d}(B_i\upharpoonright_{L_i})$ and $u_i\upharpoonright_{L_i} = v'_i$ since $u_i\upharpoonright_{\mathsf{d}(B_i)} = v_i$
- $\mathsf{d}(C_i) \cap K_i = R_i = \mathsf{d}(C_i) \cap {w_i}^{-1}(L)$ since $u_i\upharpoonright_{\mathsf{d}(C_i)} = v \circ w_i$ and $L = v^{-1}(J)$, hence $\mathsf{d}(C_i) \cap K_i = \mathsf{d}(C_i\upharpoonright_{R_i})$, and also $u_i\upharpoonright_{L_i} = v' \circ w'_i$.

It follows that $\mathsf{d}(A_i\upharpoonright_{K_i}) = L_i + R_i$, and, setting $u'_i = u_i\upharpoonright_{K_i}$, we have $u'_i\upharpoonright_{L_i} = v'_i$ and $u'_i\upharpoonright_{R_i} = v' \circ w'_i$. Hence we have a proof $\pi'$ of $(A_i\upharpoonright_{K_i}^{u'_i})_{i=1}^n \vdash A\upharpoonright_J$ such that $\underline{\pi'}_{\vec{x}} = \left(\underline{\mu'}_{\vec{x}}\right) \underline{\rho'}_{\vec{x}} = \left(\underline{\mu}_{\vec{x}}\right) \underline{\rho}_{\vec{x}} = \underline{\pi}_{\vec{x}}$ as contended. □

Though substitution lemmas are usually trivial, the $\mathsf{LJ}(I)$ substitution lemma requires some care in its statement and proof[6].

**Lemma 4 (Substitution).** *Assume that* $(A_j^{u_j})_{j=1}^n \vdash A$ *with a proof* $\mu$ *and that, for some* $i \in \{1, \dots, n\}$, $(B_j^{v_j})_{j=1}^{n-1} \vdash A_i$ *with a proof* $\rho$*. Then there is a proof* $\pi$ *of* $(C_j^{w_j})_{j=1}^{n-1} \vdash A$ *such that* $\underline{\pi}_{(\vec{x})\setminus i} = \underline{\mu}_{\vec{x}}\left[\underline{\rho}_{(\vec{x})\setminus i}/x_i\right]$ *as soon as for each* $j = 1, \dots, n-1$, $\mathsf{d}(C_j) = \mathsf{d}(A_{\mathsf{s}(j,i)}) + \mathsf{d}(B_j)$ *for each* $j = 1, \dots, n-1$ *(remember that this requires also that* $\mathsf{d}(A_{\mathsf{s}(j,i)}) \cap \mathsf{d}(B_j) = \emptyset$*) with:*

[6] We use notations introduced in Section 1, especially for $\mathsf{s}(j,i)$.

– $C_j\restriction_{\mathsf{d}(A_{\mathsf{s}(j,i)})} = A_{\mathsf{s}(j,i)}$ *and* $w_j\restriction_{\mathsf{d}(A_{\mathsf{s}(j,i)})} = u_{\mathsf{s}(j,i)}$
– $C_j\restriction_{\mathsf{d}(B_j)} = B_j$ *and* $w_j\restriction_{\mathsf{d}(B_j)} = u_i \circ v_j$.

*Proof.* By induction on the proof $\mu$. Assume that $\mu$ is an axiom, so that there is a $k \in \{1,\dots,n\}$ such that $A = u_{k*}(A_k)$, $u_k$ is a bijection and $\mathsf{d}(A_j) = \emptyset$ for all $j \neq k$. In that case we have $\underline{\mu}_{\vec{x}} = x_k$. There are two subcases to consider. Assume first that $k = i$. By Lemma 2 there is a proof $\rho'$ of $(B_j^{u_i\circ v_j})_{j=1}^{n-1} \vdash u_{i*}(A_i)$ such that $\underline{\rho'}_{(\vec{x})\setminus i} = \underline{\rho}_{(\vec{x})\setminus i}$. We have $C_j = B_j$ and $w_j = u_i \circ v_j$ for $j = 1,\dots,n-1$, so that $\rho'$ is a proof of $(C_j^{w_j})_{j=1}^{n-1} \vdash A$, so we take $\pi = \rho'$ and equation $\underline{\pi}_{(\vec{x})\setminus i} = \underline{\mu}_{\vec{x}}\left[\underline{\rho}_{(\vec{x})\setminus i}/x_i\right]$ holds since $\underline{\mu}_{\vec{x}} = x_i$. Assume next that $k \neq i$, then $\mathsf{d}(A_i) = \emptyset$ and hence $\mathsf{d}(B_j) = \emptyset$ (and $v_j = 0_\emptyset$) for $j = 1,\dots,n-1$. Therefore $C_j = A_{\mathsf{s}(j,i)}$ and $w_j = v_{\mathsf{s}(j,i)}$ for $j = 1,\dots,n-1$. So our target sequent $(C_j^{w_j})_{j=1}^{n-1} \vdash A$ can also be written $(A_{\mathsf{s}(j,i)}^{u_{\mathsf{s}(j,i)}})_{j=1}^{n-1} \vdash u_{k*}(A_k)$ and is provable by a proof $\pi$ such that $\underline{\pi}_{(\vec{x})\setminus i} = x_k$ as contended.

Assume now that $\mu$ is a $\Rightarrow$-intro, that is $A = (A_{n+1} \Rightarrow_{u_{n+1}} A')$ and $\mu$ is

$$\dfrac{\begin{array}{c}\theta\\ (A_j^{u_j})_{j=1}^{n+1} \vdash A'\end{array}}{(A_j^{u_j})_{j=1}^{n} \vdash A}$$

We set $B_n = A_{n+1}\restriction_\emptyset$ and of course $v_{n+1} = 0_{\mathsf{d}(A)}$. Then we have a proof $\rho'$ of $(B_j^{v_j})_{j=1}^{n} \vdash A_i$ such that $\underline{\rho'}_{(\vec{x})\setminus i,x_{n+1}} = \underline{\rho}_{(\vec{x})\setminus i}$ by Lemma 1. We set $C_n = A_{n+1}$ and $w_n = u_{n+1}$. Then by inductive hypothesis applied to $\theta$ we have a proof $\pi^0$ of $(C_j^{w_j})_{j=1}^{n} \vdash A'$ which satisfies $\underline{\pi^0}_{(\vec{x})\setminus i,x_{n+1}} = \underline{\theta}_{\vec{x},x_{n+1}}\left[\underline{\rho}_{(\vec{x})\setminus i}/x_i\right]$ and applying a $\Rightarrow$-introduction rule we get a proof $\pi$ of $(C_j^{w_j})_{j=1}^{n-1} \vdash A$ such that $\underline{\pi}_{(\vec{x})\setminus i} = \lambda x_{n+1}\,(\underline{\theta}_{\vec{x},x_{n+1}}\left[\underline{\rho}_{(\vec{x})\setminus i}/x_i\right]) = \underline{\mu}_{\vec{x}}\left[\underline{\rho}_{(\vec{x})\setminus i}/x_i\right]$ as expected.

Assume last that the proof $\mu$ ends with

$$\dfrac{\begin{array}{c}\varphi\\ (E_j^{s_j})_{j=1}^{n} \vdash E \Rightarrow_s A\end{array} \qquad \begin{array}{c}\psi\\ (F_j^{t_j})_{j=1}^{n} \vdash E\end{array}}{(A_j^{u_j})_{j=1}^{n} \vdash A}$$

with $\mathsf{d}(A_j) = \mathsf{d}(E_j) + \mathsf{d}(F_j)$, $A_j\restriction_{\mathsf{d}(E_j)} = E_j$, $A_j\restriction_{\mathsf{d}(F_j)} = F_j$, $u_j\restriction_{\mathsf{d}(E_j)} = s_j$ and $u_j\restriction_{\mathsf{d}(F_j)} = s \circ t_j$, for $j = 1,\dots,n$. And we have $\underline{\mu}_{\vec{x}} = \left(\underline{\varphi}_{\vec{x}}\right)\underline{\psi}_{\vec{x}}$. The idea is to "share" the substituting proof $\rho$ of $(B_j^{v_j})_{j=1}^{n} \vdash A_i$ among $\varphi$ and $\psi$ according to what they need, as specified by the formulas $E_i$ and $F_i$. So we write $\mathsf{d}(B_j) = L_j + R_j$ where $L_j = {v_j}^{-1}(\mathsf{d}(E_i))$ and $R_j = {v_j}^{-1}(\mathsf{d}(F_i))$ and by Lemma 3 we have two proofs $\rho^L$ of $(B_j\restriction_{L_j}^{v_j^L})_{j=1}^{n-1} \vdash E_i$ and $(B_j\restriction_{R_j}^{v_j^R})_{j=1}^{n-1} \vdash F_i$ where we set $v_j^L = v_j\restriction_{L_j}$ and $v_j^R = v_j\restriction_{R_j}$, obtained from $\rho$ by restriction. These proofs satisfy $\underline{\rho^L}_{(\vec{x})\setminus i} = \underline{\rho^R}_{(\vec{x})\setminus i} = \underline{\rho}_{(\vec{x})\setminus i}$.

Now we want to apply the inductive hypothesis to $\varphi$ and $\rho^L$, in order to get a proof of the sequent $(G_j^{w_j^L})_{j=1}^{n-1} \vdash E \Rightarrow_s A$ where $G_j = C_j{\upharpoonright}_{\mathsf{d}(E_{\mathsf{s}(j,i)})+L_j}$ (observe indeed that $\mathsf{d}(E_{\mathsf{s}(j,i)}) \subseteq \mathsf{d}(A_{\mathsf{s}(j,i)})$ and $L_j \subseteq \mathsf{d}(B_j)$ and hence are disjoint by our assumption that $\mathsf{d}(C_j) = \mathsf{d}(A_{\mathsf{s}(j,i)}) + \mathsf{d}(B_j)$) and $w_j^L = w_j{\upharpoonright}_{\mathsf{d}(E_{\mathsf{s}(j,i)})+L_j}$. With these definitions, and by our assumptions about $C_j$ and $w_j$, we have for all $j = 1, \ldots, n-1$

$$\begin{aligned}
G_j{\upharpoonright}_{\mathsf{d}(E_{\mathsf{s}(j,i)})} &= C_j{\upharpoonright}_{\mathsf{d}(A_{\mathsf{s}(j,i)})}{\upharpoonright}_{\mathsf{d}(E_{\mathsf{s}(j,i)})} = A_{\mathsf{s}(j,i)}{\upharpoonright}_{\mathsf{d}(E_{\mathsf{s}(j,i)})} = E_{\mathsf{s}(j,i)} \\
w_j^L{\upharpoonright}_{\mathsf{d}(E_{\mathsf{s}(j,i)})} &= w_j{\upharpoonright}_{\mathsf{d}(A_{\mathsf{s}(j,i)})}{\upharpoonright}_{\mathsf{d}(E_{\mathsf{s}(j,i)})} = u_{\mathsf{s}(j,i)}{\upharpoonright}_{\mathsf{d}(E_{\mathsf{s}(j,i)})} = s_{\mathsf{s}(j,i)} \\
G_j{\upharpoonright}_{L_j} &= C_j{\upharpoonright}_{\mathsf{d}(B_j)}{\upharpoonright}_{L_j} = B_j{\upharpoonright}_{L_j} \\
w_j^L{\upharpoonright}_{L_j} &= w_j{\upharpoonright}_{\mathsf{d}(B_j)}{\upharpoonright}_{L_j} = (u_i \circ v_j){\upharpoonright}_{L_j} = u_i{\upharpoonright}_{\mathsf{d}(E_i)} \circ v_j^L = s_i \circ v_j^L\,.
\end{aligned}$$

Therefore the inductive hypothesis applies yielding a proof $\varphi'$ of $(G_j^{w_j^L})_{j=1}^{n-1} \vdash E \Rightarrow_s A$ such that $\underline{\varphi'}_{(\vec{x})\setminus i} = \underline{\varphi}_{\vec{x}}\left[\underline{\rho^L}_{(\vec{x})\setminus i}/x_i\right] = \underline{\varphi}_{\vec{x}}\left[\underline{\rho}_{(\vec{x})\setminus i}/x_i\right]$.

Next we want to apply the inductive hypothesis to $\psi$ and $\rho^R$, in order to get a proof of the sequent $(H_j^{r_j})_{j=1}^{n-1} \vdash E$ where, for $j = 1, \ldots, n-1$, $H_j = C_j{\upharpoonright}_{\mathsf{d}(F_{\mathsf{s}(j,i)})+R_j}$ (again $\mathsf{d}(F_{\mathsf{s}(j,i)}) \subseteq \mathsf{d}(A_{\mathsf{s}(j,i)})$ and $R_j \subseteq \mathsf{d}(B_j)$ are disjoint by our assumption that $\mathsf{d}(C_j) = \mathsf{d}(A_{\mathsf{s}(j,i)}) + \mathsf{d}(B_j)$) and $r_j$ is defined by $r_j{\upharpoonright}_{\mathsf{d}(F_{\mathsf{s}(j,i)})} = t_{\mathsf{s}(j,i)}$ and $r_j{\upharpoonright}_{R_j} = t_i \circ v_j^R$. Remember indeed that $v_j^R : R_j \to \mathsf{d}(F_i)$ and $t_i : \mathsf{d}(F_i) \to \mathsf{d}(E)$. We have

$$\begin{aligned}
H_j{\upharpoonright}_{\mathsf{d}(F_{\mathsf{s}(j,i)})} &= C_j{\upharpoonright}_{\mathsf{d}(A_{\mathsf{s}(j,i)})}{\upharpoonright}_{\mathsf{d}(F_{\mathsf{s}(j,i)})} = A_{\mathsf{s}(j,i)}{\upharpoonright}_{\mathsf{d}(F_{\mathsf{s}(j,i)})} = F_{\mathsf{s}(j,i)} \\
H_j{\upharpoonright}_{R_j} &= C_j{\upharpoonright}_{\mathsf{d}(B_j)}{\upharpoonright}_{R_j} = B_j{\upharpoonright}_{R_j}
\end{aligned}$$

and hence by inductive hypothesis there is a proof $\psi'$ of $(H_j^{r_j})_{j=1}^{n-1} \vdash E$ such that $\underline{\psi'}_{(\vec{x})\setminus i} = \underline{\psi}_{\vec{x}}\left[\underline{\rho^R}_{(\vec{x})\setminus i}/x_i\right] = \underline{\psi}_{\vec{x}}\left[\underline{\rho}_{(\vec{x})\setminus i}/x_i\right]$.

To end the proof of the lemma, it will be sufficient to prove that we can apply a $\Rightarrow$-elimination rule to the sequents $(G_j^{w_j^L})_{j=1}^{n-1} \vdash E \Rightarrow_s A$ and $(H_j^{r_j})_{j=1}^{n-1} \vdash E$ in order to get a proof $\pi$ of the sequent $(C_j^{w_j})_{j=1}^{n-1} \vdash A$. Indeed, the proof $\pi$ obtained in that way will satisfy $\underline{\pi}_{(\vec{x})\setminus i} = \left(\underline{\varphi'}_{(\vec{x})\setminus i}\right)\underline{\psi'}_{(\vec{x})\setminus i} = \underline{\mu}_{\vec{x}}\left[\underline{\rho}_{(\vec{x})\setminus i}/x_i\right]$. Let $j \in \{1, \ldots, n-1\}$. We have $C_j{\upharpoonright}_{\mathsf{d}(G_j)} = G_j$ and $C_j{\upharpoonright}_{\mathsf{d}(H_j)} = H_j$ simply because $G_j$ and $H_j$ are defined by restricting $C_j$. Moreover $\mathsf{d}(G_j) = \mathsf{d}(E_{\mathsf{s}(j,i)}) + L_j$ and $\mathsf{d}(H_j) = \mathsf{d}(F_{\mathsf{s}(j,i)}) + R_j$. Therefore $\mathsf{d}(G_j) \cap \mathsf{d}(H_j) = \emptyset$ and

$$\mathsf{d}(C_j) = \mathsf{d}(A_{\mathsf{s}(j,i)}) + \mathsf{d}(B_j) = \mathsf{d}(E_{\mathsf{s}(j,i)}) + \mathsf{d}(F_{\mathsf{s}(j,i)}) + L_j + R_j = \mathsf{d}(G_j) + \mathsf{d}(H_j)\,.$$

We have $w_j{\upharpoonright}_{\mathsf{d}(G_j)} = w_j^L$ by definition of $w_j^L$ as $w_j{\upharpoonright}_{\mathsf{d}(E_{\mathsf{s}(j,i)})+L_j}$. We have

$$\begin{aligned}
w_j{\upharpoonright}_{\mathsf{d}(H_j)}{\upharpoonright}_{\mathsf{d}(F_{\mathsf{s}(j,i)})} &= w_j{\upharpoonright}_{\mathsf{d}(A_{\mathsf{s}(j,i)})}{\upharpoonright}_{\mathsf{d}(F_{\mathsf{s}(j,i)})} = u_{\mathsf{s}(j,i)}{\upharpoonright}_{\mathsf{d}(F_{\mathsf{s}(j,i)})} \\
&= s \circ t_{\mathsf{s}(j,i)} = (s \circ r_j){\upharpoonright}_{\mathsf{d}(F_{\mathsf{s}(j,i)})} \\
w_j{\upharpoonright}_{\mathsf{d}(H_j)}{\upharpoonright}_{R_j} &= w_j{\upharpoonright}_{\mathsf{d}(B_j)}{\upharpoonright}_{R_j} = (u_i \circ v_j){\upharpoonright}_{R_j} \\
&= u_i{\upharpoonright}_{\mathsf{d}(F_i)} \circ v_j^R = s \circ t_i \circ v_j^R = s \circ r_j{\upharpoonright}_{R_j} = (s \circ r_j){\upharpoonright}_{R_j}
\end{aligned}$$

and therefore $w_j\restriction_{\mathsf{d}(H_j)} = s \circ r_j$ as required. □

We shall often use the two following consequences of the Substitution Lemma.

**Lemma 5.** *Given a proof $\mu$ of $(A_j^{u_j})_{j=1}^n \vdash A$ and a proof $\rho$ of $B^v \vdash A_i$ (for some $i \in \{1,\dots,n\}$), there is a proof $\pi$ of $(A_j^{u_j})_{j=1}^{i-1}, B^{u_i \circ v}, (A_j^{u_j})_{j=i+1}^n \vdash A$ such that $\underline{\pi}_{\vec{x}} = \underline{\mu}_{\vec{x}}\left[\underline{\rho}_{x_i}/x_i\right]$*

*Proof.* By weakening we have a proof $\mu'$ of $(A_j^{u_j})_{j=1}^i, B\restriction_{\emptyset}^{0_{\mathsf{d}(A)}}, (A_j^{u_j})_{j=i+1}^n \vdash A$ such that $\underline{\mu'}_{\vec{x}} = \underline{\mu}_{(\vec{x})\setminus i+1}$ (where $\vec{x}$ is a list of pairwise distinct variables of length $n{+}1$), as well as a proof $\rho'$ of $(A_j\restriction_{\emptyset}^{0_{\mathsf{d}(A_i)}})_{j=1}^i, B^v, (A_j\restriction_{\emptyset}^{0_{\mathsf{d}(A_i)}})_{j=i+1}^n \vdash A_i$ such that $\underline{\rho'}_{\vec{x}} = \underline{\rho}_{x_{i+1}}$. By Lemma 4, we have a proof $\pi'$ of $(A_j^{u_j})_{j=1}^{i-1}, B^{u_i \circ v}, (A_j^{u_j})_{j=i+1}^n \vdash A$ which satisfies $\underline{\pi'}_{(\vec{x})\setminus i} = \underline{\mu'}_{\vec{x}}\left[\underline{\rho'}_{(\vec{x})\setminus i}/x_i\right] = \underline{\mu}_{\vec{x}}\left[\underline{\rho}_{x_i}/x_i\right]$. □

**Lemma 6.** *Given a proof $\mu$ of $A^v \vdash B$ and a proof $\rho$ of $(A_j^{u_j})_{j=1}^n \vdash A$, there is a proof $\pi$ of $(A_j^{v \circ u_j})_{j=1}^n \vdash B$ such that $\underline{\pi}_{\vec{x}} = \underline{\mu}_x\left[\underline{\rho}_{\vec{x}}/x\right]$.*

The proof is similar to the previous one.

If $A$ and $B$ are formulas such that $\underline{A} = \underline{B}$, $\mathsf{d}(A) = \mathsf{d}(B)$ and $\langle A\rangle = \langle B\rangle$, we say that $A$ and $B$ are similar and we write $A \sim B$. One fundamental property of our deduction system is that two formulas which represent the same family of intersection types are logically equivalent.

**Theorem 1.** *If $A \sim B$ then $A^{\mathsf{Id}} \vdash B$ with a proof $\pi$ such that $\underline{\pi}_x \sim_\eta x$.*

*Proof.* Assume that $A = \alpha[f]$, then we have $B = A$ and $A^{\mathsf{Id}} \vdash B$ is an axiom.

Assume that $A = (C \Rightarrow_u D)$ and $B = (E \Rightarrow_v F)$. We have $D \sim F$ and hence $D^{\mathsf{Id}} \vdash F$ with a proof $\rho$ such that $\underline{\rho}_x \sim_\eta x$. And there is a bijection $w : \mathsf{d}(E) \to \mathsf{d}(C)$ such that $w_*(E) \sim C$ and $u \circ w = v$. By inductive hypothesis we have a proof $\mu$ of $w_*(E)^{\mathsf{Id}} \vdash C$ such that $\underline{\mu}_y \sim_\eta y$, and hence using the axiom $E^w \vdash w_*(E)$ and Lemma 5 we have a proof $\mu'$ of $E^w \vdash C$ such that $\underline{\mu'}_x = \underline{\mu}_x$.

There is a proof $\pi^1$ of $(C \Rightarrow_u D)^{\mathsf{Id}}, C^u \vdash D$ such that $\underline{\pi^1}_{x,y} = (x)\,y$ (consider the two axioms $(C \Rightarrow_u D)^{\mathsf{Id}}, C\restriction_{\emptyset}^{0_{\mathsf{d}(D)}} \vdash C \Rightarrow_u D$ and $(C \Rightarrow_u D)\restriction_{\emptyset}^{0_{\mathsf{d}(C)}}, C^{\mathsf{Id}} \vdash C$ and use a $\Rightarrow$-elimination rule). So by Lemma 5 there is a proof $\pi^2$ of $(C \Rightarrow_u D)^{\mathsf{Id}}, E^{u \circ w} \vdash D$, that is of $(C \Rightarrow_u D)^{\mathsf{Id}}, E^v \vdash D$, such that $\underline{\pi^2}_{x,y} = (x)\,\underline{\mu}_y$. Applying Lemma 6 we get a proof $\pi^3$ of $(C \Rightarrow_u D)^{\mathsf{Id}}, E^v \vdash F$ such that $\underline{\pi^3}_{x,y} = \underline{\rho}_z\left[(x)\,\underline{\mu}_y/z\right]$. We get the expected proof $\pi$ by a $\Rightarrow$-introduction rule so that $\underline{\pi}_x = \lambda y\,\underline{\rho}_z\left[(x)\,\underline{\mu}_y/z\right]$. By inductive hypothesis $\underline{\pi}_x \sim_\eta x$. □

### 3.4 Relation between intersection types and $\mathsf{LJ}(I)$

Now we explain the precise connection between non-idempotent intersection types and our logical system $\mathsf{LJ}(I)$. This connection consists of two statements:

- the first one means that any proof of $\mathsf{LJ}(I)$ can be seen as a typing derivation in non-idempotent intersection types (soundness)
- and the second one means that any non-idempotent intersection typing can be seen as a derivation in $\mathsf{LJ}(I)$ (completeness).

**Theorem 2 (Soundness).** *Let $\pi$ be a deduction tree of the sequent $(A_i^{u_i})_{i=1}^n \vdash B$ and $\overrightarrow{x}$ a sequence of $n$ pairwise distinct variables. Then the $\lambda$-term $\underline{\pi}_{\overrightarrow{x}}$ satisfies $(x_i : \langle A_i^{u_i}\rangle_j : \underline{A_i})_{i=1}^n \vdash \underline{\pi}_{\overrightarrow{x}} : \langle B\rangle_j : \underline{B}$ in the intersection type system, for each $j \in \mathsf{d}(B)$.*

*Proof.* We prove the first part by induction on $\pi$ (in the course of this induction, we recall the precise definition of $\underline{\pi}_{\overrightarrow{x}}$). If $\pi$ is the proof

$$\frac{q \neq i \Rightarrow \mathsf{d}(A_q) = \emptyset \text{ and } u_i \text{ is a bijection}}{(A_q^{u_q})_{q=1}^n \vdash u_{i*}(A_i)}$$

(so that $B = u_{i*}(A_i)$) then $\underline{\pi}_{\overrightarrow{x}} = x_i$. We have $\langle A_q^{u_q}\rangle_j = [\,]$ if $q \neq i$, $\langle A_i^{u_i}\rangle_j = [\,\langle A_i\rangle_{u_i^{-1}(j)}\,]$ and $\langle u_{i*}(A_i)\rangle_j = \langle A_i\rangle_{u_i^{-1}(j)}$. It follows that $(x_q : \langle A_q^{u_q}\rangle_j : \underline{A_q})_{q=1}^n \vdash x_i : \langle B\rangle_j : \underline{B}$ is a valid axiom in the intersection type system.

Assume that $\pi$ is the proof

$$\frac{\begin{array}{c}\pi^0\\ A_1^{u_1}, \dots, A_n^{u_n}, A^u \vdash B\end{array}}{A_1^{u_1}, \dots, A_n^{u_n} \vdash A \Rightarrow_u B}$$

where $\pi^0$ is the proof of the premise of the last rule of $\pi$. By inductive hypothesis the $\lambda$-term $\underline{\pi^0}_{\overrightarrow{x},x}$ satisfies $(x_i : \langle A_i^{u_i}\rangle_j : \underline{A_i})_{i=1}^n, x : \langle A^u\rangle_j : \underline{A} \vdash \underline{\pi^0}_{\overrightarrow{x},x} : \langle B\rangle_j : \underline{B}$ from which we deduce $(x_i : \langle A_i^{u_i}\rangle_j : \underline{A_i})_{i=1}^n \vdash \lambda x^{\underline{A}}\,\underline{\pi^0}_{\overrightarrow{x},x} : (\langle A^u\rangle_j, \langle B\rangle_j) : \underline{A} \Rightarrow \underline{B}$ which is the required judgment since $\underline{\pi}_{\overrightarrow{x}} = \lambda x^{\underline{A}}\,\underline{\pi^0}_{\overrightarrow{x},x}$ and $(\langle A_i^{u_i}\rangle_j, \langle B\rangle_j) = \langle A \Rightarrow_u B\rangle_j$ as easily checked.

Assume last that $\pi$ ends with

$$\frac{\begin{array}{c}\pi^1\\ C_1^{u_1}, \dots, C_n^{u_n} \vdash A \Rightarrow_u B\end{array} \qquad \begin{array}{c}\pi^2\\ D_1^{v_1}, \dots, D_n^{v_n} \vdash A\end{array}}{E_1^{w_1}, \dots, E_n^{w_n} \vdash B}$$

with: for each $i = 1, \dots, n$ there are two disjoint sets $L_i$ and $R_i$ such that $\mathsf{d}(E_i) = L_i + R_i$, $C_i = E_i\lceil_{L_i}$, $D_i = E_i\lceil_{R_i}$, $w_i\lceil_{L_i} = u_i$, and $w_i\lceil_{R_i} = u \circ v_i$.

Let $j \in \mathsf{d}(B)$. By inductive hypothesis, the judgment $(x_i : \langle C_i^{u_i}\rangle_j : \underline{C_i})_{i=1}^n \vdash \underline{\pi^1}_{\overrightarrow{x}} : \langle A \Rightarrow_u B\rangle_j : \underline{A} \Rightarrow \underline{B}$ is derivable in the intersection type system. Let $K_j = u^{-1}(\{j\})$, which is a finite subset of $\mathsf{d}(A)$. By inductive hypothesis again, for each $k \in K_j$ we have $(x_i : \langle D_i^{u_i}\rangle_k : \underline{D_i})_{i=1}^n \vdash \underline{\pi^2}_{\vec{x}} : \langle A\rangle_k : \underline{A}$. Now observe that $\langle A \Rightarrow_u B\rangle_j = ([\,\langle A\rangle_k \mid k \in K_j\,], \langle B\rangle_j)$ so that

$$(x_i : \langle C_i^{u_i}\rangle_j + \sum_{k\in K_j} \langle D_i^{u_i}\rangle_k : \underline{E_i})_{i=1}^n \vdash \left(\underline{\pi^1}_{\vec{x}}\right) \underline{\pi^2}_{\vec{x}} : \langle B\rangle_j : \underline{B}$$

is derivable in intersection types (remember that $\underline{C_i} = \underline{D_i} = \underline{E_i}$). Since $\underline{\pi}_{\vec{x}} = \left(\underline{\pi^1}_{\vec{x}}\right) \underline{\pi^2}_{\vec{x}}$ it will be sufficient to prove that

$$\langle E_i^{w_i}\rangle_j = \langle C_i^{u_i}\rangle_j + \sum_{k\in K_j} \langle D_i^{v_i}\rangle_k\,. \tag{2}$$

For this, since $\langle E_i^{w_i}\rangle_j = [\,\langle E_i\rangle_l \mid w_i(l) = j\,]$, consider an element $l$ of $\mathsf{d}(E_i)$ such that $w_i(l) = j$. There are two possibilities: (1) either $l \in L_i$ and in that case we know that $\langle E_i\rangle_l = \langle C_i\rangle_l$ since $E_i\lceil_{L_i} = C_i$ and moreover we have $u_i(l) = w_i(l) = j$ (2) or $l \in R_i$. In that case we have $\langle E_i\rangle_l = \langle D_i\rangle_l$ since $E_i\lceil_{R_i} = D_i$. Moreover $u(v_i(l)) = w_i(l) = j$ and hence $v_i(l) \in K_j$. Therefore

$$[\,\langle E_i\rangle_l \mid l \in L_i \text{ and } w_i(l) = j\,] = [\,\langle C_i\rangle_l \mid u_i(l) = j\,] = \langle C_i^{u_i}\rangle_j$$
$$[\,\langle E_i\rangle_l \mid l \in R_i \text{ and } w_i(l) = j\,] = [\,\langle D_i\rangle_l \mid v_i(l) \in K_j\,] = \sum_{k\in K_j} \langle D_i^{v_i}\rangle_k$$

and (2) follows. □

**Theorem 3 (Completeness).** *Let $J \subseteq I$. Let $M$ be a $\lambda$-term and $x_1, \dots, x_n$ be pairwise distinct variables, such that $(x_i : m_i^j : \sigma_i)_{i=1}^n \vdash M : b_j : \tau$ in the intersection type system for all $j \in J$. Let $A_1, \dots, A_n$ and $B$ be formulas and let $u_1, \dots, u_n$ be almost injective functions such that $u_i : \mathsf{d}(A_i) \to J = \mathsf{d}(B)$. Assume also that $\underline{A_i} = \sigma_i$ for each $i = 1, \dots, n$ and that $\underline{B} = \tau$. Last assume that, for all $j \in J$, one has $\langle B\rangle_j = b_j$ and $\langle A_i^{u_i}\rangle_j = m_i^j$ for $i = 1, \dots, n$. Then the judgment $(A_i^{u_i})_{i=1}^n \vdash B$ has a proof $\pi$ such that $\underline{\pi}_{\vec{x}} \sim_\eta M$.*

*Proof.* By induction on $M$. Assume first that $M = x_i$ for some $i \in \{1, \dots, n\}$. Then we must have $\tau = \sigma_i$, $m_q^j = [\,]$ for $q \neq i$ and $m_i^j = [\,b_j\,]$ for all $j \in J$. Therefore $\mathsf{d}(A_q) = \emptyset$ and $u_q$ is the empty function for $q \neq i$, $u_i$ is a bijection $\mathsf{d}(A_i) \to J$ and $\forall k \in \mathsf{d}(A_i)\ \langle A_i\rangle_k = b_{u_i(k)}$, in other words $u_{i*}(A_i) \sim B$. By Theorem 1 we know that the judgment $(u_{i*}(A_i))^{\mathsf{Id}} \vdash B$ is provable in $\mathsf{LJ}(I)$ with a proof $\rho$ such that $\underline{\rho}_x \sim_\eta x$. We have a proof $\theta$ of $(A_i^{u_i})_{i=1}^n \vdash u_{i*}(A_i)$ which consists of an axiom so that $\underline{\theta}_{\vec{x}} = x_i$ and hence by Lemma 6 we have a proof $\pi$ of $(A_i^{u_i})_{i=1}^n \vdash B$ such that $\underline{\pi}_{\vec{x}} = \underline{\rho}_x\,[\underline{\theta}_{\vec{x}}/x] \sim_\eta x_i$.

Assume that $M = \lambda x^\sigma N$, that $\tau = (\sigma \Rightarrow \varphi)$ and that we have a family of deductions (for $j \in J$) of $(x_i : m_i^j : \sigma_i)_{i=1}^n \vdash M : (m^j, c_j) : \sigma \Rightarrow \varphi$ with $b_j = (m^j, c_j)$ and the premise of this conclusion in each of these deductions is $(x_i : m_i^j : \sigma_i)_{i=1}^n, x : m^j : \sigma \vdash N : c_j : \varphi$. We must have $B = (C \Rightarrow_u D)$ with $\underline{D} = \varphi$, $\underline{C} = \sigma$, $\mathsf{d}(D) = J$, $u : \mathsf{d}(C) \to \mathsf{d}(D)$ almost injective, $\langle D\rangle_j = c_j$ and

$[\,\langle C\rangle_k \mid k \in \mathsf{d}(C) \text{ and } u(k)=j\,] = m^j$, that is $\langle C^u\rangle_j = m^j$, for each $j \in J$. By inductive hypothesis we have a proof $\rho$ of $(A_i^{u_i})_{i=1}^n, C^u \vdash D$ such that $\underline{\rho}_{\vec{x},x} \sim_\eta N$ from which we obtain a proof $\pi$ of $(A_i^{u_i})_{i=1}^n \vdash C \Rightarrow_u D$ such that $\underline{\pi}_{\vec{x}} = \lambda x^\sigma\, \underline{\rho}_{\vec{x},x} \sim_\eta M$ as expected.

Assume last that $M = (N)\,P$ and that we have a $J$-indexed family of deductions $(x_i : m_i^j : \sigma_i)_{i=1}^n \vdash M : b_j : \tau$ . Let $A_1, \dots, A_n$, $u_1, \dots, u_n$ and $B$ be $\mathsf{LJ}(I)$ formulas and almost injective functions as in the statement of the theorem.

Let $j \in J$. There is a finite set $L_j \subseteq I$ and multisets $m_i^{j,0}$, $(m_i^{j,l})_{l \in L_j}$ such that we have deductions[7] of $(x_i : m_i^{j,0} : \sigma_i)_{i=1}^n \vdash N : ([\,a_l^j \mid l \in L_j\,], b_j) : \sigma \Rightarrow \tau$ and, for each $l \in L_j$, of $(x_i : m_i^{j,l} : \sigma_i)_{i=1}^n \vdash P : a_l^j : \sigma$ with

$$m_i^j = m_i^{j,0} + \sum_{l \in L_j} m_i^{j,l}\,. \tag{3}$$

We assume the finite sets $L_j$ to be pairwise disjoint (this is possible because $I$ is infinite) and we use $L$ for their union. Let $u : L \to J$ be the function which maps $l \in L$ to the unique $j$ such that $l \in L_j$, this function is almost injective. Let $A$ be an $\mathsf{LL}(J)$ formula such that $\underline{A} = \sigma$, $\mathsf{d}(A) = L$ and $\langle A\rangle_l = a_l^{u(l)}$; such a formula exists by Proposition 1.

Let $i \in \{1, \dots, n\}$. For each $j \in J$ we know that

$$[\,\langle A_i\rangle_r \mid r \in \mathsf{d}(A_i) \text{ and } u_i(r) = j\,] = m_i^j = m_i^{j,0} + \sum_{l \in L_j} m_i^{j,l}$$

and hence we can split the set $\mathsf{d}(A_i) \cap {u_i}^{-1}(\{j\})$ into disjoint subsets $R_i^{j,0}$ and $(R_i^{j,l})_{l \in L_j}$ in such a way that

$$[\,\langle A_i\rangle_r \mid r \in R_i^{j,0}\,] = m_i^{j,0} \quad \text{and} \quad \forall l \in L_j\ [\,\langle A_i\rangle_r \mid r \in R_i^{j,l}\,] = m_i^{j,l}\,.$$

We set $R_i^0 = \bigcup_{j \in J} R_i^{j,0}$; observe that this is a disjoint union because $R_i^{j,0} \subseteq {u_i}^{-1}(\{j\})$. Similarly we define $R_i^1 = \bigcup_{l \in L} R_i^{u(l),l}$ which is a disjoint union for the following reason: if $l, l' \in L$ satisfy $u(l) = u(l') = j$ then $R_i^{j,l}$ and $R_i^{j,l'}$ have been chosen disjoint and if $u(l) = j$ and $u(l') = j'$ with $j \neq j'$ we have $R_i^{j,l} \subseteq {u_i}^{-1}\{j\}$ and $R_i^{j',l'} \subseteq {u_i}^{-1}(\{j'\})$. Let $v_i : R_i^1 \to L$ be defined by: $v_i(r)$ is the unique $l \in L$ such that $r \in R_i^{u(l),l}$. Since each $R_i^{j,l}$ is finite the function $v_i$ is almost injective. Moreover $u \circ v_i = u_i\restriction_{R_i^1}$.

We use $u_i'$ for the restriction of $u_i$ to $R_i^0$ so that $u_i' : R_i^0 \to J$. By inductive hypothesis we have $((A_i\restriction_{R_i^0})^{u_i'})_{i=1}^n \vdash A \Rightarrow_u B$ with a proof $\mu$ such that $\underline{\mu}_{\vec{x}} \sim_\eta N$. Indeed $[\,\langle A_i\restriction_{R_i^0}\rangle_r \mid r \in R_i^0 \text{ and } u_i'(r) = j\,] = m_i^{j,0}$ and $\langle A \Rightarrow_u B\rangle_j = ([\,a_l^j \mid u(l) = j\,], b_j)$ for each $j \in J$. For the same reason we have $((A_i\restriction_{R_i^1})^{v_i})_{i=1}^n \vdash A$ with a proof $\rho$ such that $\underline{\rho}_{\vec{x}} \sim_\eta P$. Indeed for each $l \in L = \mathsf{d}(A)$ we have

[7] Notice that our $\lambda$-calculus is in *Church style* and hence the type $\sigma$ is uniquely determined by the sub-term $N$ of $M$.

$[\langle A_i\lceil_{R_i^1}\rangle_r \mid v_i(r) = l\,] = m_i^{j,l}$ and $\langle A\rangle_l = a_l^j$ where $j = u(l)$. By an application rule we get a proof $\pi$ of $(A_i^{u_i})_{i=1}^n \vdash B$ such that $\underline{\pi}_{\overrightarrow{x}} = \left(\underline{\mu}_{\overrightarrow{x}}\right) \underline{\rho}_{\overrightarrow{x}} \sim_\eta (N)\, P = M$ as contended. □

## 4 The untyped Scott case

Since intersection types usually apply to the pure $\lambda$-calculus, we move now to this setting by choosing in **Rel**$_!$ the set $\mathsf{R}_\infty$ as model of the pure $\lambda$-calculus. The $\mathsf{R}_\infty$ intersection typing system has the elements of $\mathsf{R}_\infty$ as types, and the typing rules involve sequents of shape $(x_i : m_i)_{i=1}^n \vdash M : a$ where $m_i \in \mathcal{M}_{\mathrm{fin}}(\mathsf{R}_\infty)$ and $a \in \mathsf{R}_\infty$.

We use $\Lambda$ for the set of terms of the pure $\lambda$-calculus, and $\Lambda_\Omega$ as the pure $\lambda$-calculus extended with a constant $\Omega$ subject to the two following $\rightsquigarrow_\omega$ reduction rules: $\lambda x\, \Omega \rightsquigarrow_\omega \Omega$ and $(\Omega)\, M \rightsquigarrow_\omega \Omega$. We use $\sim_{\eta\omega}$ for the least congruence on $\Lambda_\Omega$ which contains $\rightsquigarrow_\eta$ and $\rightsquigarrow_\omega$ and similarly for $\sim_{\beta\eta\omega}$. We define a family $(\mathcal{H}(x))_{x\in\mathcal{V}}$ of subsets of $\Lambda_\Omega$ minimal such that, for any sequence $\overrightarrow{x} = (x_1, \dots, x_n)$ and $\overrightarrow{y} = (y_1, \dots, y_k)$ such that $\overrightarrow{x}, \overrightarrow{y}$ is repetition-free, and for any terms $M_i \in \mathcal{H}(x_i)$ (for $i = 1, \dots, n$), one has $\lambda\overrightarrow{x}\, \lambda\overrightarrow{y}\, (x)\, M_1 \cdots M_n\, O_1 \cdots O_l \in \mathcal{H}(x)$ where $O_j \sim_\omega \Omega$ for $j = 1, \dots, l$. Notice that $x \in \mathcal{H}(x)$.

The typing rules of $\mathsf{R}_\infty$ are

$$\frac{}{x_1 : [\,], \dots, x_i : [\,a\,], \dots, x_n : [\,] \vdash x_i : a} \qquad \frac{\Phi, x : m \vdash M : a}{\Phi \vdash \lambda x\, M : (m, a)}$$

$$\frac{\Phi \vdash M : ([\,a_1, \dots, a_k\,], b) \qquad (\Phi_j \vdash N : a_j)_{j=1}^k}{\Phi + \sum_{j=1}^k \Phi_j \vdash (M)\, N : b}$$

where we use the following convention: when we write $\Phi + \Psi$ it is assumed that $\Phi$ is of shape $(x_i : m_i)_{i=1}^n$ and $\Psi$ is of shape $(x_i : p_i)_{i=1}^n$, and then $\Phi + \Psi$ is $(x_i : m_i + p_i)_{i=1}^n$. This typing system is just a "proof-theoretic" rephrasing of the denotational semantics of the terms of $\Lambda_\Omega$ in $\mathsf{R}_\infty$.

**Proposition 2.** *Let $M, M' \in \Lambda_\Omega$ and $\overrightarrow{x} = (x_1, \dots, x_n)$ be a list of pairwise distinct variables containing all the free variables of $M$ and $M'$. Let $m_i \in \mathcal{M}_{\mathrm{fin}}(\mathsf{R}_\infty)$ for $i = 1, \dots, n$ and $b \in \mathsf{R}_\infty$. If $M \sim_{\beta\eta\omega} M'$ then $(x_i : m_i)_{i=1}^n \vdash M : b$ iff $(x_i : m_i)_{i=1}^n \vdash M' : b$.*

### 4.1 Formulas

We define the associated formulas as follows, each formula $A$ being given together with $\mathsf{d}(A) \subseteq I$ and $\langle A\rangle \in \mathsf{R}_\infty^{\mathsf{d}(A)}$.

- If $J \subseteq I$ then $\varepsilon_J$ is a formula with $\mathsf{d}(\varepsilon_J) = J$ and $\langle\varepsilon_J\rangle_j = \mathsf{e}$ for $j \in J$
- and if $A$ and $B$ are formulas and $u : \mathsf{d}(A) \to \mathsf{d}(B)$ is almost injective then $A \Rightarrow_u B$ is a formula with $\mathsf{d}(A \Rightarrow_u B) = \mathsf{d}(B)$ and $\langle A \Rightarrow_u B\rangle_j = ([\,\langle A\rangle_k \mid u(k) = j\,], \langle B\rangle_j) \in \mathsf{R}_\infty$.

We can consider that there is a type $\mathsf{o}$ of pure $\lambda$-terms interpreted as $\mathsf{R}_\infty$ in $\mathbf{Rel}_!$, such that $(\mathsf{o} \Rightarrow \mathsf{o}) = \mathsf{o}$, and then for any formula $A$ we have $\underline{A} = \mathsf{o}$.

Operations of restriction and relocation of formulas are the same as in Section 3 (setting $\varepsilon_J \upharpoonright_K = \varepsilon_{J \cap K}$) and satisfy the same properties, for instance $\langle A \upharpoonright_K \rangle = \langle A \rangle \upharpoonright_K$ and one sets $u_*(\varepsilon_J) = \varepsilon_K$ if $u : J \to K$ is a bijection.

The deduction rules are exactly the same as those of Section 3, plus the axiom $\vdash \varepsilon_\emptyset$. With any deduction $\pi$ of $(A_i^{u_i})_{i=1}^n \vdash B$ and sequence $\overrightarrow{x} = (x_1, \dots, x_n)$ of pairwise distinct variables, we can associate a *pure* $\underline{\pi}_{\overrightarrow{x}} \in \Lambda_\Omega$ defined exactly as in Section 3 (just drop the types associated with variables in abstractions). If $\pi$ consists of an instance of the additional axiom, we set $\underline{\pi}_{\overrightarrow{x}} = \Omega$.

**Lemma 7.** *Let $A, A_1, \dots, A_n$ be a formula such that $\mathsf{d}(A) = \mathsf{d}(A_i) = \emptyset$. Then $(A_i^{0_\emptyset})_{i=1}^n \vdash A$ is provable by a proof $\pi$ which satisfies $\underline{\pi}_{x_1,\dots,x_k} \sim_\omega \Omega$.*

The proof is a straightforward induction on $A$ using the additional axiom, Lemma 1 and the observations that if $\mathsf{d}(B \Rightarrow_u C) = \emptyset$ then $u = 0_\emptyset$.

One can easily define a size function $\mathsf{sz} : \mathsf{R}_\infty \to \mathbb{N}$ such that $\mathsf{sz}(\mathsf{e}) = 0$ and $\mathsf{sz}([\, a_1, \dots, a_k \,], a) = \mathsf{sz}(a) + \sum_{i=1}^k (1 + \mathsf{sz}(a_i))$. First we have to prove an adapted version of Proposition 1; here it will be restricted to finite sets.

**Proposition 3.** *Let $J$ be a* finite *subset of $I$ and $f \in \mathsf{R}_\infty^J$. There is a formula $A$ such that $\mathsf{d}(A) = J$ and $\langle A \rangle = f$.*

*Proof.* Observe that, since $J$ is finite, there is an $N \in \mathbb{N}$ such that $\forall j \in J\ \forall q \in \mathbb{N}\ q \geq N \Rightarrow f(j)_q = [\,]$ (remember that $f(j) \in \mathcal{M}_{\mathrm{fin}}(\mathsf{R}_\infty)^{\mathbb{N}}$). Let $N(f)$ be the least such $N$. We set $\mathsf{sz}(f) = \sum_{j \in J} \mathsf{sz}(f(j))$ and the proof is by induction on $(\mathsf{sz}(f), N(f))$ lexicographically.

If $\mathsf{sz}(f) = 0$ this means that $f(j) = \mathsf{e}$ for all $j \in J$ and hence we can take $A = \varepsilon_J$. Assume that $\mathsf{sz}(f) > 0$, one can write[8] $f(j) = (m_j, a_j)$ with $m_j \in \mathcal{M}_{\mathrm{fin}}(\mathsf{R}_\infty)$ and $a_j \in \mathsf{R}_\infty$ for each $j \in J$. Just as in the proof of Proposition 1 we choose a set $K$, a function $g : K \to \mathsf{R}_\infty$ and an almost injective function $u : K \to J$ such that $m_j = [\, g(k) \mid u(k) = j \,]$. The set $K$ is finite since $J$ is and we have $\mathsf{sz}(g) < \mathsf{sz}(f)$ because $\mathsf{sz}(f) > 0$. Therefore by inductive hypothesis there is a formula $B$ such that $\mathsf{d}(B) = K$ and $\langle B \rangle = g$. Let $f' : J \to \mathsf{R}_\infty$ defined by $f'(j) = a_j$, we have $\mathsf{sz}(f') \leq \mathsf{sz}(f)$ and $N(f') < N(f)$ and hence by inductive hypothesis there is a formula $C$ such that $\langle C \rangle = f$. We set $A = (B \Rightarrow_u C)$ which satisfies $\langle A \rangle = f$ as required. ☐

Theorem 1 still holds up to some mild adaptation. First notice that $A \sim B$ simply means now that $\mathsf{d}(A) = \mathsf{d}(B)$ and $\langle A \rangle = \langle B \rangle$.

**Theorem 4.** *If $A$ and $B$ are such that $A \sim B$ then $A^{\mathsf{Id}} \vdash B$ with a proof $\pi$ which satisfies $\underline{\pi}_x \in \mathcal{H}(x)$.*

[8] This is also possible if $\mathsf{sz}(f) = 0$ actually.

*Proof.* By induction on the sum of the sizes of $A$ and $B$. Assume that $A = \varepsilon_J$ so that $\mathsf{d}(B) = J$ and $\forall j \in J\ \langle B\rangle_j = \mathsf{e}$. There are two cases as to $B$. In the first case $B$ is of shape $\varepsilon_K$ but then we must have $K = J$ and we can take for $\pi$ an axiom so that $\underline{\pi}_x = x \in \mathcal{H}(x)$. Otherwise we have $B = (C \Rightarrow_u D)$ with $\mathsf{d}(D) = J$, $\forall j \in J\ \langle D\rangle_j = \mathsf{e}$ and $\mathsf{d}(C) = \emptyset$, so that $u = 0_J$. We have $A \sim D$ and hence by inductive hypothesis we have a proof $\rho$ of $A^{\mathsf{Id}} \vdash D$ such that $\underline{\rho}_x \in \mathcal{H}(x)$. By weakening and $\Rightarrow$-introduction we get a proof $\pi$ of $A^{\mathsf{Id}} \vdash B$ which satisfies $\underline{\pi}_x = \lambda y\, \underline{\rho}_x \in \mathcal{H}(x)$.

Assume that $A = (C \Rightarrow_u D)$. If $B = \varepsilon_J$ then we must have $\mathsf{d}(C) = \emptyset$, $u = 0_J$ and $D \sim B$ and hence by inductive hypothesis we have a proof $\rho$ of $D^{\mathsf{Id}} \vdash B$ such that $\underline{\rho}_x \in \mathcal{H}(x)$. By Lemma 7 there is a proof $\theta$ of $\vdash C$ such that $\underline{\theta} \sim_\omega \Omega$. Hence there is a proof $\pi$ of $A^{\mathsf{Id}} \vdash B$ such that $\underline{\pi}_x = \underline{\rho}_y\,[(x)\,\underline{\theta}/y] \in \mathcal{H}(x)$.

Assume last that $B = (E \Rightarrow_v F)$, then we must have $D \sim F$ and there must be a bijection $w : \mathsf{d}(E) \to \mathsf{d}(C)$ such that $u \circ w = v$ and $w_*(E) \sim C$. We reason as in the proof of Lemma 1: by inductive hypothesis we have a proof $\rho$ of $D^{\mathsf{Id}} \vdash F$ and a proof $\mu$ of $w_*(E)^{\mathsf{Id}} \vdash C$ from which we build a proof $\pi$ of $A^{\mathsf{Id}} \vdash B$ such that $\underline{\pi}_x = \lambda y\, \underline{\rho}_z \left[(x)\,\underline{\mu}_y/z\right] \in \mathcal{H}(x)$ by inductive hypothesis. □

**Theorem 5 (Soundness).** *Let $\pi$ be a deduction tree of $A_1^{u_1}, \dots, A_n^{u_n} \vdash B$ and $\vec{x}$ a sequence of $n$ pairwise distinct variables. Then the λ-term $\underline{\pi}_{\vec{x}} \in \Lambda_\Omega$ satisfies $(x_i : \langle A_i^{u_i}\rangle_j)_{i=1}^n \vdash \underline{\pi}_{\vec{x}} : \langle B\rangle_j$ in the $\mathsf{R}_\infty$ intersection type system, for each $j \in \mathsf{d}(B)$.*

The proof is exactly the same as that of Theorem 2, dropping all simple types.

For all λ-term $M \in \Lambda$, we define $\mathcal{H}_\Omega(M)$ as the least subset of element of $\Lambda_\Omega$ such that:

- if $O \in \Lambda_\Omega$ and $O \sim_\omega \Omega$ then $O \in \mathcal{H}_\Omega(M)$ for all $M \in \Lambda$
- if $M = x$ then $\mathcal{H}(x) \subseteq \mathcal{H}_\Omega(M)$
- if $M = \lambda y\, N$ and $N' \in \mathcal{H}_\Omega(N)$ then $\lambda y\, N' \in \mathcal{H}_\Omega(M)$
- if $M = (N)\,P$, $N' \in \mathcal{H}_\Omega(N)$ and $P' \in \mathcal{H}_\Omega(P)$ then $(N')\,P' \in \mathcal{H}_\Omega(M)$.

The elements of $\mathcal{H}_\Omega(M)$ can probably be seen as approximates of $M$.

**Theorem 6 (Completeness).** *Let $J \subseteq I$ be* finite. *Let $M \in \Lambda_\Omega$ and $x_1, \dots, x_n$ be pairwise distinct variables, such that $(x_i : m_i^j)_{i=1}^n \vdash M : b_j$ in the $\mathsf{R}_\infty$ intersection type system for all $j \in J$. Let $A_1, \dots, A_n$ and $B$ be formulas and let $u_1, \dots, u_n$ be almost injective functions such that $u_i : \mathsf{d}(A_i) \to J = \mathsf{d}(B)$. Assume also that, for all $j \in J$, one has $\langle B\rangle_j = b_j$ and $\langle A_i^{u_i}\rangle_j = m_i^j$ for $i = 1, \dots, n$. Then the judgment $A_1^{u_1}, \dots, A_n^{u_n} \vdash B$ has a proof $\pi$ such that $\underline{\pi}_{\vec{x}} \in \mathcal{H}_\Omega(M)$.*

The proof is very similar to that of Theorem 3.

## 5 Concluding remarks and acknowledgments

The results presented in this paper show that, at least in non-idempotent intersection types, the problem of knowing whether all elements of a given family of intersection types $(a_j)_{j\in J}$ are inhabited by a common $\lambda$-term can be reformulated logically: is it true that one (or equivalently, any) of the indexed formulas $A$ such that $\mathsf{d}(A) = J$ and $\forall j \in \ \langle A\rangle_j = a_j$ is provable in $\mathsf{LJ}(I)$? Such a strong connection between intersection and Indexed Linear Logic was already mentioned in the introduction of [2], but we never made it more explicit until now.

To conclude we propose a typed $\lambda$-calculus *à la Church* to denote proofs of the $\mathsf{LJ}(I)$ system of Section 4. The syntax of *pre-terms* is given by $s, t \ldots := x[J] \mid \lambda x : A^u\, s \mid (s)\, t$ where in $x[J]$, $x$ is a variable and $J \subseteq I$ and, in $\lambda x : A^u\, s$, $u$ is an almost injective function from $\mathsf{d}(A)$ to a set $J \subseteq I$. Given a pre-term $s$ and a variable $x$, the *domain of $x$ in $s$* is the subset $\mathsf{dom}(x, s)$ of $I$ given by $\mathsf{dom}(x, x[J]) = J$, $\mathsf{dom}(x, y[J]) = \emptyset$ if $y \neq x$, $\mathsf{dom}(x, \lambda y : A^u\, s) = \mathsf{dom}(x, s)$ (assuming of course $y \neq x$) and $\mathsf{dom}(x, (s)\, t) = \mathsf{dom}(x, s) \cup \mathsf{dom}(x, t)$. Then a pre-term $s$ is a term if any subterm of $t$ which is of shape $(s_1)\, s_2$ satisfies $\mathsf{dom}(x, s_1) \cap \mathsf{dom}(x, s_2) = \emptyset$ for all variable $x$. A typing judgment is an expression $(x_i : A_i^{u_i})_{i=1}^n \vdash s : B$ where the $x_i$'s are pairwise distinct variables, $s$ is a term and each $u_i$ is an almost injective function $\mathsf{d}(A_i) \to \mathsf{d}(B)$. The following typing rules exactly mimic the logical rules of $\mathsf{LJ}(I)$:

$$\frac{\mathsf{d}(A) = \emptyset}{((x_i : A_i^{0_\emptyset})_{i=1}^n) \vdash \Omega : A}$$

$$\frac{q \neq i \Rightarrow \mathsf{d}(A_i) = \emptyset \text{ and } u_i \text{ bijection}}{(x_q : A_q^{u_q})_{q=1}^n \vdash x_i[\mathsf{d}(A_i)] : u_{i*}(A_i)} \qquad \frac{(x_i : A_i^{u_i})_{i=1}^n, x : A^u \vdash s : B}{(x_i : A_i^{u_i})_{i=1}^n \vdash \lambda x : A^u\, s : A \Rightarrow_u B}$$

$$\frac{(x_i : A_i{\upharpoonright}_{\mathsf{dom}(x_i,s)}^{v_i})_{i=1}^n \vdash s : A \Rightarrow_u B \qquad (x_i : A_i{\upharpoonright}_{\mathsf{dom}(x_i,t)}^{w_i})_{i=1}^n \vdash t : A}{(x_i : A_i^{v_i + (u \circ w_i)})_{i=1}^n \vdash (s)\, t : B}$$

The properties of this calculus, and more specifically of its $\beta$-reduction, and its connections with the resource calculus of [9] will be explored in further work.

Another major objective will be to better understand the meaning of $\mathsf{LJ}(I)$ formulas, using ideas developed in [3] where a *phase semantics* is introduced and related to (non-uniform) coherence space semantics. In the intuitionistic present setting, it is tempting to look for Kripke-like interpretations with the hope of generalizing indexed logic beyond the (perhaps too) specific relational setting we started from.

Last, we would like to thank Luigi Liquori and Claude Stolze for many helpful discussions on intersection types and the referees for their careful reading and insightful comments and suggestions.
